# Unmasking the Dual Threat of Fentanyl and Xylazine Abuse in America

**DOI:** 10.1192/j.eurpsy.2024.463

**Published:** 2024-08-27

**Authors:** M. S. Bicho, J. M. Coelho, B. Peixoto, C. Cruz, P. Baião, I. Ferreira

**Affiliations:** Psychiatry, Hospital do Divino Espírito Santo de Ponta Delgada, Ponta Delgada, Portugal

## Abstract

**Introduction:**

The United States of America are currently facing a public health crisis characterized by the abuse of synthetic opioids, notably Fentanyl, and the veterinary sedative Xylazine. While each of these substances has been associated with significant risks, their current misuse presents a formidable challenge to healthcare professionals, law enforcement agencies and policymakers. While the opioid epidemic has long held the nation in its grip, the emergence of Xylazine as complementary agent in substance abuse has added a disturbing layer of complexity to an already terrible situation, due to its cost-cutting, an increase in its addictive properties and its ability to extend the duration of the opioid with which it is combined.

**Objectives:**

The authors intend to review the relevant and current literature in order to extend the knowledge about this condition and find the best conducts for clinical practice.

**Methods:**

Non-systematic literature review

**Results:**

Various regions of the United States are facing a troubling surge in the co-abuse of Fentanyl, a potent synthetic opioid many times more potent than morphine, and Xylazine, a veterinary sedative and muscle relaxant, particularly in urban areas. The motivations for this combination appear to vary, ranging from the enhanced euphoria to cost-saving measures, further fueling its prevalence. However, the consequences are devastating. Both substances depress the central nervous system, with a sharp increase in overdose deaths and emergency medical services are strained to their limits in responding to these crises. Law enforcement agencies are facing a daunting task in curtailing the distribution of these substances, often grappling with clandestine networks that exploit the accessibility of these drugs.

**Conclusions:**

The concurrent abuse of Fentanyl and Xylazine represents a critical public health challenge in the United States of America, demanding immediate attention and a multidisciplinary response. Failure to address this issue comprehensively will have profound implications for the well-being of individuals, families and communities across the nation. It is imperative to mobilize resources, foster interdisciplinary collaboration and develop evidence-based policies to combat this dual-threat crisis. Novel intervention strategies, including community education programs, targeted outreach efforts, and supervised consumption facilities, are urgently needed to address this complex issue.

**Disclosure of Interest:**

None Declared

